# The Efficacy of Antihypertensive Drugs and miR-632 Inhibition on Parietal Remodeling in a Model of Marfan Thoracic Aortic Aneurysm

**DOI:** 10.3390/biom16060863

**Published:** 2026-06-12

**Authors:** Sonia Terriaca, Maria Giovanna Scioli, Fabio Bertoldo, Paolo Nardi, Gian Paolo Novelli, Beatrice Belmonte, Tommaso D’Anna, Carmela Rita Balistreri, Calogera Pisano, Amedeo Ferlosio, Augusto D’Onofrio, Augusto Orlandi

**Affiliations:** 1 Anatomic Pathology, Azienda Ospedaliera Universitaria Policlinico Tor Vergata, 00133 Rome, Italy; terriacasonia093@gmail.com; 2 Anatomic Pathology, Department of Biomedicine and Prevention, Tor Vergata University, 00133 Rome, Italy; tommaso.danna@students.uniroma2.eu (T.D.); ferlosio@uniroma2.it (A.F.); orlandi@uniroma2.it (A.O.); 3 Cardiac Surgery Unit, Azienda Ospedaliera Universitaria Policlinico Tor Vergata, 00133 Rome, Italy; fabio.bertoldo@ptvonline.it; 4 Cardiac Surgery Unit, Department of Surgery, Tor Vergata University, 00133 Rome, Italy; pa.nardi4@libero.it (P.N.); adonofrio@hotmail.it (A.D.); 5 Division of Cardiology, Azienda Ospedaliera Universitaria Policlinico Tor Vergata, 00133 Rome, Italy; gianpaolo.novelli@ptvonline.it; 6 Tumor Immunology Unit, Department of Health Sciences, University of Palermo, 90134 Palermo, Italy; beatrice.belmonte@unipa.it; 7 ASP Catania-P.O. “GRAVINA E SANTO PIETRO” di Caltagirone, 95124 Catania, Italy; 8 Cellular and Molecular Laboratory, Department of Biomedicine, Neuroscience and Advanced Diagnostics (Bi.N.D.), University of Palermo, 90134 Palermo, Italy; carmelarita.balistreri@unipa.it; 9 Cardiac Surgery Unit, Department of Precision Medicine in Medical Surgical and Critical Area (Me.Pre.C.C.), University of Palermo, 90134 Palermo, Italy; calogera.pisano01@unipa.it

**Keywords:** Marfan syndrome, thoracic aortic aneurysm, carvedilol, ramipril, losartan, miR-632 inhibition, aortic wall remodeling

## Abstract

Background: Marfan syndrome (MFS) is a connective tissue disorder caused by *FBN1* mutations, leading to elastic fiber disarray and early thoracic aortic aneurysm (TAA) formation. Currently, pharmacological treatments lack specificity and only delay progression. We previously reported a specific TGFβ-driven miR-632 upregulation in MFS TAA tissues and blood causing smooth muscle cell dedifferentiation and aortic wall degeneration. This study evaluated the effects of three conventional antihypertensive drugs (β-blocker, ACE inhibitor, and sartan) on parietal remodeling, comparing them with a miR-632 inhibitor in an ex vivo TGFβ1-induced model of MFS TAA. Methods and Results: Using an ex vivo paired experimental framework based on independent biological pools of human TAA tissue, gene expression and Western blot analyses demonstrated that only losartan significantly reduced miR-632 and vascular degeneration markers. Notably, combined treatment with ramipril and carvedilol compromised losartan’s efficacy, highlighting the need for careful therapeutic selection. In this ex vivo setting, miR-632 inhibition demonstrated a promising capacity to counteract aortic remodeling, serving as a mechanistic proof-of-concept that warrants further preclinical in vivo validation. Conclusions: Our data emphasize that choosing the right treatment in MFS aortopathy requires understanding its specific impact on cellular pathways. Our findings identify losartan as the most effective standard drug in this model, while suggesting miR-632 as a potential future target to stabilize the aortic wall and, prospectively, delay surgery.

## 1. Introduction

Marfan syndrome (MFS) is a genetic condition caused by mutations in the *FBN1* gene coding for the extracellular glycoprotein fibrillin-1 [1]. MFS is associated with a high risk of early thoracic aortic aneurysm (TAA) development, in particular in the aortic root [1]. *FBN1* mutation leads to Transforming Growth Factor β (TGF-β) signaling hyperactivation with early vascular wall weakness and TAA onset [2]. Although clinical manifestations can be heterogeneous, the most life-threatening complications in MFS patients occur in the cardiovascular district with mitral valve prolapse, aortic insufficiency, aortic root dilatation and TAA dissection [3]. MFS TAA is characterized by early and severe degeneration of the aortic tunica media with disruption of extracellular matrix (ECM) homeostasis [4,5]. In particular, TGFβ hyperactivation promotes ECM remodeling through the induction of matrix metalloproteinases (MMPs), particularly MMP-9, and alterations in their endogenous inhibitors such as TIMP-1 with altered MMP-9/TIMP-1 balance with excessive degradation of structural ECM components, including elastin and collagen, thereby contributing to the progressive weakening and aneurysmatic dilation of the aortic wall [4,6].

The introduction of surgery and the use of antihypertensive drugs proved effective in the management of MFS patients [7]. In particular, recent advances in surgical and therapy improved life expectancy in MFS patients [7]. Initially, the standard-of-care for MFS patients was β-blockers (e.g., carvedilol, bisoprolol); the latter regulate systemic blood pressure and cardiac pulse wave reducing vascular tension and, consequently, the risk of root dilatation and TAA rupture [7]. Angiotensin converting enzyme inhibitors (ACEi) play an important role in the development of aortic stiffening, dilatation and rupture in MFS patients [8]. Recent advances suggest that the selective inhibition of angiotensin II type 1 receptors (AT1R) through Angiotensin Receptor Blockers (ARBs) is one of the most appropriate treatment [9]. Among ARBs, losartan is reported to prevent aortic dilation in MFS patients better than other antihypertensive drugs [10]. In some therapeutic protocols, losartan is administered in combination with β-blockers and, under medical control, also with ACE inhibitor [9,11]. Despite the recent improvement of the pharmacological strategies, MFS patients still undergo early surgery. Therefore, it is mandatory to find additional or more specific therapeutic approaches to prevent TAA progression in MFS patients. MFS TAA is characterized by an early and increased aortic remodeling compared with non-MFS ones [12]. We reported that a specific miR-632 upregulation in MFS TAA tissue and blood, induced by TGF-β signaling hyperactivation, contributes to severe and early degeneration of the aortic wall [12,13]. In particular, miR-632 deregulation induced the dedifferentiation of vascular smooth muscle cells (VSMCs) into myofibroblasts, which express extra domain A fibronectin (ED-A FN) and produce excessive collagen, leading to collagen accumulation and aortic fibrosis [13].

In the present study, we investigated the effect of antihypertensive drugs (β-blocker, ACE inhibitor and ARB) and a specific miR-632 inhibitor on the TGFβ-induced degenerative process of the aortic wall in an MFS TAA ex vivo model.

## 2. Materials and Methods

### 2.1. Patients and Aortic Sample Collection

For this study, we collected aortic tissue samples from non-MFS TAA patients (n = 6) undergoing elective surgical procedures in the Department of Cardiosurgery of Azienda Ospedaliera Universitaria Policlinico Tor Vergata of Rome. The eligibility criteria used were as follows: patients aged 30–70 years with TAAs (average of aortic diameter = 53 mm), without coronary artery disease, endocarditis, aortic dissection, renal failure, liver disease, or tumor, and with a good ejection fraction. The diameter evaluation of ascending aorta was made both preoperatively and in the operating room by transthoracic echocardiography, and transesophageal echocardiography estimations were performed as follows: estimating the dimensions of the aortic annulus, the sinuses of Valsalva, and the proximal ascending aorta (above 2.5 cm of the sinotubular junction) in the parasternal long-axis view and evaluating the dimensions of the aortic arch from the suprasternal view. All echocardiography-derived dimensions were reported according to standard EAE measurement conventions [14]. Color Doppler was used to assess the presence and severity of aortic regurgitation and stenosis. The measurement of the aortic root and ascending aorta diameters was also carried out using helical computed tomography image analysis. The surgical procedure was mostly the isolated ascending aorta replacement or the button Bentall operation [14]. All operations were performed using crystalloid or hematic cardioplegia. The Territorial Ethics Committee approved the study (Protocol “PNRR-MR1-2022-12376699”) and all the patients signed the informed consent form.

### 2.2. Ex Vivo Transfection of TAA Tissues with miR-632 Inhibitor

After surgery, small aortic samples were immediately used for the experiments. Before proceeding with the experiments, the adventitia tunica was removed and endothelium was scraped, as reported [15]. Then, small fragments of fresh aortic tunica media from six non-MFS TAA patients were adhered to the bottom of a 24- or 6-well multiwall [13]. In particular, from each of the six patients, the aortic tissue was sectioned into uniform fragments. Specifically, 3 fragments per patient were used for RNA extraction in 24-well plates, and 5 fragments per patient were allocated for protein analysis in 6-well plates. To minimize biological variability, fragments from each individual patient were distributed in parallel across all experimental groups, including antihypertensive treatments, allowing each biological donor to serve as its own internal control. To simulate the hyper-TGFβ1 signaling characteristic of Marfan syndrome, non-MFS TAA tissues were treated ex vivo with exogenous TGFβ1 using a previously validated protocol [13]. This model isolates the primary pathogenic cascade for targeted pharmacological screening while avoiding confounding genetic variables. Briefly, tissue samples were primed with TGF-β1 (10 ng/mL; Sigma Aldrich, St. Louis, MO, USA) for 24 h [13]. After 24 h, TGF-β1-enriched medium was removed and tissue fragments transfected with miRCURY locked nucleic acid (LNA)-anti-miR-632 (100 nM) and scrambled LNA (negative controls) (Qiagen, Hilden, Germany). For RNA and protein extraction, the samples were collected in four pools (two pools for scrambled LNA and two for LNA-anti-miR-632, each pool = 3 patients) 24 h and 72 h after transfection, respectively (see Table 1).

### 2.3. Ex Vivo Treatment of TAA Tissues with Antihypertensive Drugs

As reported above, the fragments were ex vivo pre-treated with TGFβ1 (with 10 ng/mL) for 24 h and after 24 h the TGFβ1-enriched medium was removed, and the fragments were treated with a non-selective β-blocker (carvedilol, Sigma Aldrich, St. Louis, MI, USA), the ACE inhibitor (ramipril, Sigma Aldrich, St. Louis, MI, USA) and ARBs (losartan, Sigma Aldrich, St. Louis, MI, USA), alone or in combination using a concentration of 10 µM, as reported [16,17,18,19]. This universal concentration was selected for the ex vivo screening to allow a direct comparison of the molecular effects of the drugs on TGFβ1-induced pathways, and to ensure optimal tissue penetration within the aortic media fragments, in line with established tissue explant experimental protocols. Stock solutions for carvedilol, ramipril, and losartan were prepared in pure methanol and subsequently diluted in the culture medium. The final concentration of methanol in all treated groups and vehicle controls was strictly maintained below 0.1% (*v*/*v*) to avoid vehicle-associated cytotoxicity. For RNA and protein extraction, the samples were collected in 2 pools (each pool = 3 patients) for each treatment after 24 and 72 h, respectively (see Table 1).

### 2.4. Gene Expression Analysis

RNA and miRNA extraction was performed using the TRI Reagent^®^ (Sigma Aldrich, St. Louis, MI, USA) according to the manufacturer’s protocols, as reported [20]. A Micro-Volume spectrophotometer (EZDROP 1000 Micro-Volume spectrophotometer, Resnova, Rome, Italy) was used to measure the RNA concentration. Then, 700 ng of RNA/miRNA were reverse-transcribed using the microScript microRNA cDNA Synthesis Kit (Norgen Biotek, Thorold, ON, Canada) or the SuperScript III (Invitrogen, Thermo Fisher Scientific, Waltham, MA, USA). Real-time PCR was carried out by using SYBR Green (BioRad, Hercules, CA, USA). We used MystiCq microRNA Primer HSA-miR-632 (Merck KGaA, Darmstadt, Germany) and Universal PCR Reverse Primer (Norgen Biotek, Thorold, ON, Canada) were used to analyze miR-632 expression. For gene expression analysis, cDNAs were amplified using specific primers for *ED-A FN*. HSA-miR-632 was normalized to the *U6* expression, while other genes were normalized to the *GAPDH* expression (see Supplemental Table S1). Changes in target gene expression were calculated using the comparative ΔΔCT method [21]. Fold change was considered significant for values > 2.0 and <0.5. Analyses were performed in triplicate.

### 2.5. Western Blot Analysis

Total protein extraction was performed on non-MFS TAA tunica media that had been treated/transfected [13]. Briefly, the tissues were homogenized and resuspended in RIPA buffer (Sigma Aldrich, St. Louis, MI, USA) with protease inhibitor cocktail (Sigma Aldrich, St. Louis, MI, USA) and phosphatase inhibitors (Sigma Aldrich, St. Louis, MI, USA). Protein concentration was determined by a spectrophotometric assay (BioRad, Hercules, CA, USA). A total of 70 μg of protein was separated using 8% polyacrylamide gel and then transferred from the gel to a filter. The latter was incubated with specific mouse monoclonal anti-ED-A FN antibody (1:200, Santa Cruz Biotechnology, Dallas, TX, USA), Rabbit Polyclonal anti-MMP-9 (1:300, Proteintech, Rosemont, IL, USA), and Rabbit Monoclonal anti-TIMP-1 (1:200, Abcam, Cambridge, UK). Normalization was performed using mouse polyclonal anti-α-tubulin (1:500, Sigma Aldrich, St. Louis, MI, USA). Detection and quantification were carried out using the ECL Reagent (BioRad, Hercules, CA, USA) and LAS-4000 (Fujifilm, Tokyo, Japan). The intensity of the bands was analyzed by ImageJ software (version 1.50i, National Institutes of Health, Bethesda, MD, USA).

### 2.6. Statistical Analysis

Continuous variables are expressed as mean ± SEM. To account for the biological pooling framework, with Pool A and Pool B treated as blocking factors, a two-way Analysis of Variance (ANOVA) without replication was performed. Since the omnibus F-test revealed a statistically significant effect, post hoc pairwise comparisons were subsequently conducted using Fisher’s protected least significant difference (LSD) test. A *p*-value < 0.05 was considered statistically significant. Statistical analyses were performed with SPSS software (version 23, IBM, SPSS Statistics, Chicago, IL, USA ).

## 3. Results

### 3.1. Losartan Is the Most Effective in Reducing miR-632 Levels and Myofibroblastic Dedifferentiation in TGFβ1-Treated TAA Tunica Media

**Figure 1:**
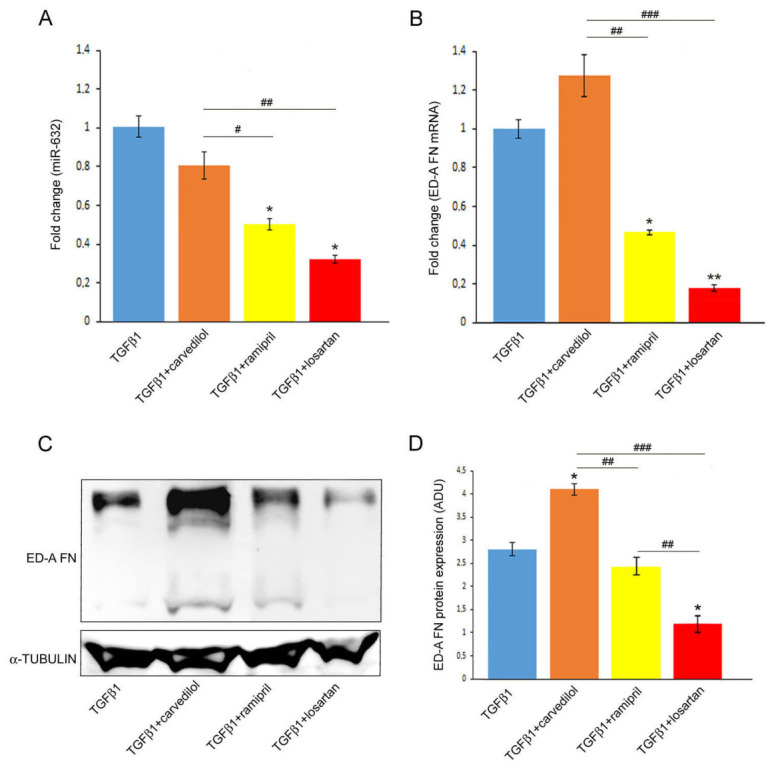
Losartan is the most effective drug in reducing miR-632 and the fibrotic marker ED-A FN expression. Gene expression analysis shows a significant downregulation of miR-632 (**A**) and *ED-A FN* (**B**) in TGFβ1-primed TAA tunica media treated with losartan compared with control (only TGFβ1; *p* < 0.01 and *p* < 0.001, respectively), carvedilol (*p* < 0.01 and *p* < 0.001, respectively) and ramipril groups (*p* < 0.05 and *p* < 0.01, respectively). Representative blots (**C**) and densitometric analysis (**D**) display a significant reduction in ED-A FN by losartan compared with the control (*p* < 0.05), carvedilol (*p* < 0.01) and ramipril groups (*p* < 0.05). Carvedilol treatment increases ED-A FN expression compared with the TGFβ1 control group (*p* = 0.05). The data represent the mean ± SEM of two independent biological pools (Pool A and Pool B), each combining tissues from three distinct patients (n = 6). A two-way Analysis of Variance (ANOVA) without replication followed by Fisher’s LSD post hoc test: * and ** indicate *p* < 0.01 and *p* < 0.001, drugs vs. TGFβ1, respectively. #, ## and ### indicate *p* < 0.05, *p* < 0.01 and *p* < 0.001 drugs vs. los, respectively. Original western blots can be found at Supplementary Materials.

In order to analyze the effects of antihypertensive drugs on miR-632 expression and on VSMC phenotypic switching into myofibroblasts in non-MFS TAA, we pre-treated tunica media tissue fragments with TGFβ1 that induces miR-632 upregulation and represents a well-established model of MFS TAA [13]. TGFβ1-pre-treated TAA fragments were successively treated with antihypertensive drugs: carvedilol, ramipril and losartan. Gene expression analysis revealed that losartan was the most effective treatment, significantly downregulating miR-632 levels (*p* < 0.01 vs. TGFβ1; Figure 1A) and demonstrating a potent inhibitory effect on *ED-A FN* at both the transcriptional and protein levels (*p* < 0.001 vs. TGFβ1; Figure 1B–D). In comparison, ramipril showed a significant inhibitory effect only on miR-632 and *ED-A FN* mRNA expression but less than losartan (*p* < 0.01 vs. TGFβ1; Figure 1A,B) whereas its impact was lost at the protein level (Figure 1C,D). Conversely, carvedilol failed to provide protection against TGFβ-induced miR-632 overexpression, instead exacerbating the profibrotic response (*p* < 0.01; Figure 1C,D).

### 3.2. Combined Antihypertensive Treatment Reduces Losartan Inhibition of miR-632 and ED-A FN in TAA Tissues

**Figure 2:**
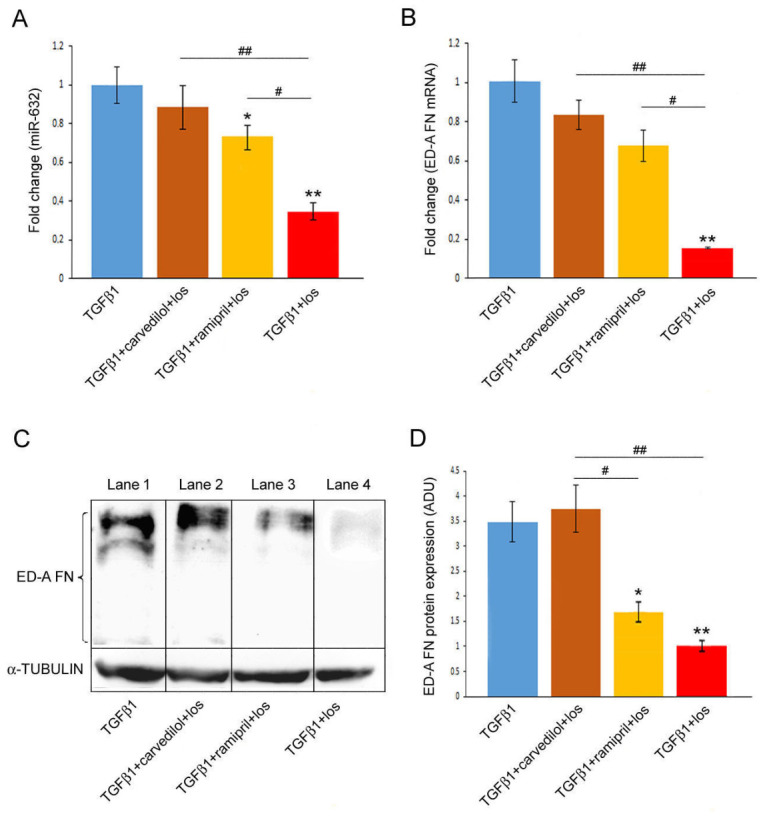
Losartan compromises its inhibitory activity on miR-632 and ED-A FN in combination with other carvedilol and ramipril. Gene expression analysis shows a significant reduction in miR-632 (**A**) and *ED-A FN* (**B**) in TGFβ1-primed TAA tissues treated with losartan alone (los) compared with combined treatments (*p* < 0.05 and *p* < 0.01 vs. ramipril + los; *p* < 0.05 and *p* < 0.01 vs. carvedilol + los, respectively). Representative blots (**C**) and densitometric analysis (**D**) of ED-A FN protein expression that confirm the results reported above. The data represent the mean ± SEM of two independent biological pools (Pool A and Pool B), each combining tissues from three distinct patients (n = 6). A two-way Analysis of Variance (ANOVA) without replication followed by Fisher’s LSD post hoc test: * and ** indicate *p* < 0.05 and *p* < 0.01, drugs vs. TGFβ1, respectively. # and ## indicate *p* < 0.05 and *p* < 0.01, combined drugs vs. losartan monotreatment, respectively. Abbreviation: los = losartan. Original western blots can be found at Supplementary Materials.

Since losartan can be administered to MFS patients in combination with β-blocker and, in some cases, with ACEi (under medical supervision), we analyzed the effects of those combinations on miR-632 levels and ED-A FN expression in ex vivo TGFβ1-stimulated TAA tunica media tissues. Our results highlighted that the potent inhibitory effect of losartan is notably attenuated when used in combination with other drugs. Specifically, the co-administration of carvedilol and losartan did not significantly reduce miR-632 and ED-A FN levels compared to losartan monotreatment (*p* < 0.01; Figure 2A–D). Similarly, although the ramipril + losartan group showed lower expression levels than the TGFβ1 control (*p* < 0.05), its efficacy was less than losartan alone (*p* < 0.05). Overall, those findings suggest that combining these pharmacological treatments does not provide a synergistic benefit; rather, it compromises the maximal efficacy of losartan on miR-632 and myofibroblastic dedifferentiation in this ex vivo TGFβ-treated TAA model.

### 3.3. Antihypertensive Drugs Differently Impact MMP-9 and TIMP-1 Expression in TGFβ-Treated TAA Tunica Media

**Figure 3:**
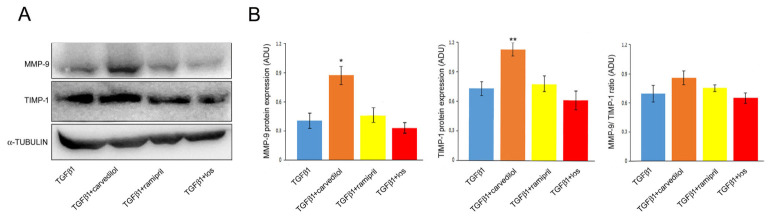
Antihypertensive drugs show differential impact on aortic proteolytic markers. Representative blots (**A**) and densitometric analysis (**B**) display that the carvedilol exposure of TGFβ1-pre-treated TAA tunica media increases MMP-9 and TIMP-1 protein levels, but not the MMP-9/TIMP-1 protein ratio compared with TGFβ1 control groups. The data represent the mean ± SEM of two independent biological pools (Pool A and Pool B), each combining tissues from three distinct patients (n = 6). A two-way Analysis of Variance (ANOVA) without replication followed by Fisher’s LSD post hoc test: * and ** indicate *p* < 0.05 and *p* < 0.01. Abbreviation: los = losartan. Original western blots can be found at Supplementary Materials.

**Figure 4:**
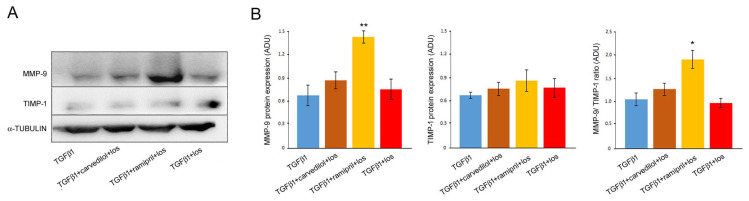
Ramipril used in combination with losartan increases the expression of MMP-9 and MMP-9/TIMP-1 ratio. Representative blots (**A**) and densitometric analysis (**B**) display that losartan used in combination with ramipril affects the MMP-9 protein level as well as the MMP-9/TIMP-1 protein ratio compared with the control TGFβ1 and losartan monotreatment groups. The data represent the mean ± SEM of two independent biological pools (Pool A and Pool B), each combining tissues from three distinct patients (n = 6). A two-way Analysis of Variance (ANOVA) without replication followed by Fisher’s LSD post hoc test: * and ** indicate *p* < 0.01 and *p* < 0.001. Abbreviation: los = losartan. Original western blots can be found at Supplementary Materials.

Since MFS TAA is characterized by extensive tissue remodeling, we evaluated the impact of different antihypertensive treatments on key remodeling markers, specifically MMP-9 and TIMP-1. As shown in Figure 3A,B, in TGFβ-treated TAA tunica media fragments, carvedilol significantly upregulated MMP-9 and TIMP-1 expression (*p* < 0.05 and *p* < 0.01, respectively). Conversely, losartan induced a slight, though non-significant, reduction in MMP-9 levels. Despite these individual changes, the MMP-9/TIMP-1 ratio remained unaffected by any of the treatments. Furthermore, we investigated whether combined treatments of losartan with either carvedilol or ramipril influenced those markers. As reported in Figure 4, the combined treatment of losartan with ramipril led to a significant increase in MMP-9 protein expression compared to TGFβ1 and losartan monotreatment (*p* < 0.001; Figure 4), consequently leading to a significantly higher MMP-9/TIMP-1 ratio (*p* < 0.01). In contrast, the addition of carvedilol did not induce significant changes in the expression of MMP-9 or TIMP-1, nor did it alter their relative ratio.

### 3.4. The Specific Inhibition of miR-632 Is the Most Effective Strategy for Reducing Aortic Wall Degeneration Markers in TGFβ-Stimulated TAA Tunica Media 

**Figure 5:**
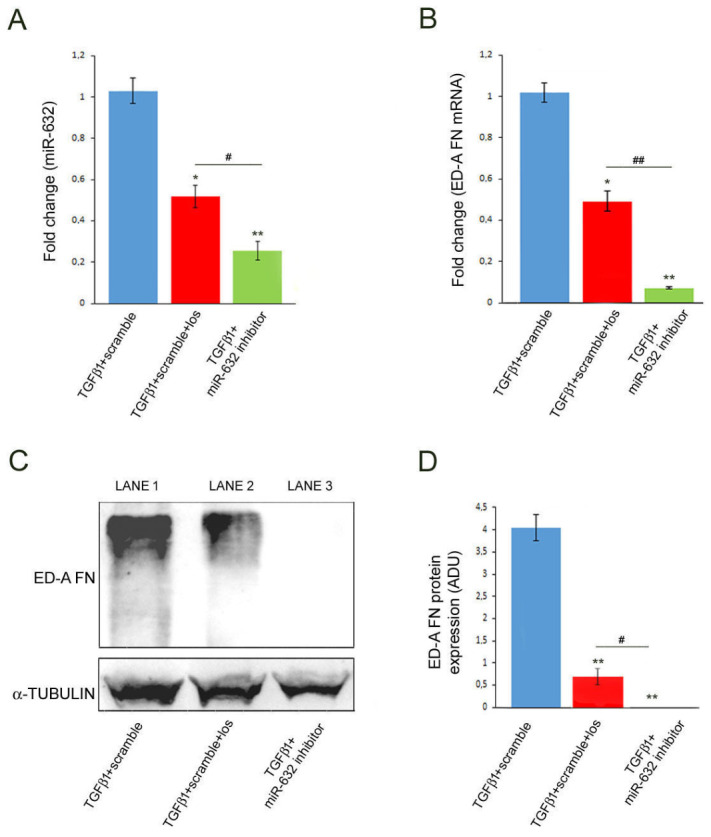
Specific miR-632 inhibition is most effective strategy at reducing the expression of fibrotic marker ED-A FN. Gene expression analysis shows a significant and potent downregulation of miR-632 (**A**) and *ED-A FN* (**B**) in pre-treated TAA tissues transfected with miR-632 inhibitor compared with the control (TGFβ1 + scramble) (*p* < 0.001) or losartan (scramble + los) (*p* < 0.01). Representative blots (**C**) and densitometric analysis (**D**) confirm gene expression results. The data represent the mean ± SEM of two independent biological pools (Pool A and Pool B), each combining tissues from three distinct patients (n = 6). A two-way Analysis of Variance (ANOVA) without replication followed by Fisher’s LSD post hoc test: * and ** indicate *p* < 0.05 and *p* < 0.01 vs. TGFβ1 + scramble, respectively. # and ## indicate *p* < 0.05 and *p* < 0.01, miR-632 inhibitor vs. scramble + los. Abbreviation: los = losartan. Original western blots can be found at Supplementary Materials.

**Figure 6:**
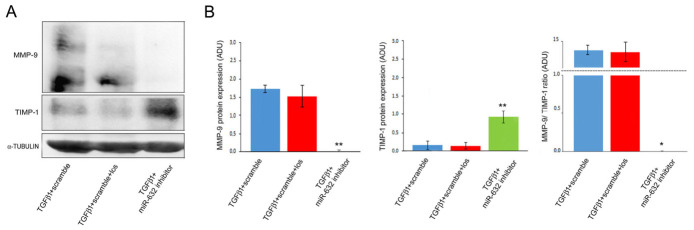
The specific inhibition of miR-632 is most effective strategy at counteracting aortic remodeling process. Representative blots and (**A**) and densitometric analysis (**B**) display that the treatment of TAA tissues (primed with TGFβ1) with miR-632 inhibitor increases TIMP-1 protein levels and reduces MMP-9 levels (*p* < 0.01) as well as the MMP-9/TIMP-1 protein ratio (*p* < 0.001) compared with TGFβ1 + scramble or TGFβ1 + scramble + los. The data represent the mean ± SEM of two independent biological pools (Pool A and Pool B), each combining tissues from three distinct patients (n = 6). A two-way Analysis of Variance (ANOVA) without replication followed by Fisher’s LSD post hoc test: * and ** indicated *p* < 0.02 and *p* < 0.01, respectively. Abbreviation: los = losartan. Original western blots can be found at Supplementary Materials.

Following our comparative analysis of antihypertensive agents, we further evaluated the efficacy of losartan, the clinical standard for MFS patients, in comparison with a specific miR-632 inhibitor. In TGFβ-treated TAA tunica media fragments, losartan treatment successfully reduced the expression of miR-632 and ED-A FN at both the transcriptional and protein levels (*p* < 0.05 and *p* < 0.01 vs. TGFβ1; Figure 5A–D). However, the specific inhibition of miR-632 resulted in a more pronounced downregulation of those markers (*p* < 0.01 vs. TGFβ1). Regarding the proteolytic balance, while losartan maintained it unaltered (Figure 6A,B), miR-632 inhibitor exerted a stronger impact by reducing MMP-9 and upregulating TIMP-1 protein expression (*p* < 0.01; Figure 6A,B), thereby strongly decreasing the MMP-9/TIMP-1 ratio (*p* < 0.02; Figure 6A,B).

## 4. Discussion

The pharmacological management of MFS TAA primarily focuses on mitigating the hemodynamic and molecular triggers of aortic wall remodeling. Our findings provide a detailed comparison of how conventional antihypertensive agents—carvedilol, ramipril, and losartan—and novel epigenetic targets, such as miR-632, modulate the ECM degradation that characterizes MFS TAA. Previous studies from our group demonstrated that miR-632 expression is significantly upregulated in both the TAA tissue and blood of MFS patients and plays a key role in promoting aortic wall degeneration [13,20]. In particular, in MFS aorta, TGFβ hyperactivation induced miR-632 upregulation, leading to aortic fibrosis, ECM disorganization and progressive weakening of the aortic wall [13]. In this context, our findings demonstrated that among conventional antihypertensive drugs, losartan, used as a monotreatment, is the most effective in reducing the fibrotic markers (i.e., miR-632 expression and ED-A FN). Those findings support the hypothesis of a complex molecular crosstalk between the Renin-Angiotensin System and the TGFβ pathway. In fact, previous reports demonstrated that angiotensin II type 1 receptor blockade by losartan attenuates TGFβ signaling, a central pathway in MFS aortopathy pathogenesis [22,23,24]. Indeed, losartan was shown to inhibit both canonical and non-canonical TGF-β pathways, thereby limiting downstream profibrotic responses [25,26]. Concerning the proteolytic profile, our results suggest that losartan actively contributes to maintaining ECM homeostasis rather than inducing a robust inhibition of remodeling markers. In our experimental model, losartan treatment showed a slight trend toward reducing MMP-9 levels, while leaving the overall MMP-9/TIMP-1 ratio substantially unchanged. Those findings suggest that while losartan, through its blockade of the AT1 receptor and partial modulation of the TGFβ pathway, contributes to the stabilization of the tunica media, it does not exert a predominant effect on the proteolytic balance.

In contrast, ramipril, acting upstream as an ACE inhibitor, may fail to completely prevent local Angiotensin II synthesis likely due to alternative pathways like chymases [27], explaining its more modest effect on miR-632 and ED-A FN expression failing to translate this reduction to the protein level. Regarding the proteolytic profile, ramipril monotreatment did not significantly modulate MMP-9 or TIMP-1 expression. In our experimental conditions, the protein levels remained largely stationary, with no detectable shift in the MMP-9/TIMP-1 ratio. This lack of effect suggests that, in this specific MFS TAA model, ACE inhibition alone is insufficient to alter the proteolytic profile of the tunica media. This observation is consistent with the presence of alternative local pathways for Angiotensin II synthesis, such as chymases as already reported [27], which may render the proteolytic markers unresponsive to ramipril treatment, thereby maintaining the remodeling markers at their baseline levels. Concerning the effects of carvedilol, we did not document a significant modulation of miR-632 levels compared to the TGFβ1-treated control. However, despite this lack of epigenetic regulation, we observed that carvedilol promoted a further increase in ED-A FN expression. This discrepancy suggests that carvedilol may negatively influence aortic remodeling through pathways independent of miR-632. The upregulation of ED-A FN by a non-selective beta-blocker could be linked to its complex interaction with the TGFβ signaling axis. While carvedilol is clinically established for its role in reducing hemodynamic stress, several studies provide conflicting evidence regarding the anti-fibrotic potential of β-blockers. Some studies suggest that β-adrenergic ligands may paradoxically influence extracellular matrix deposition under specific pathological conditions [28,29]. Our observations align with these discrepancies: while some research highlights the protective effects of β-blockade [30], others report minimal or no impact depending on the experimental model utilized [31]. Notably, to the best of our knowledge, no previous studies have specifically addressed the influence of carvedilol on VSMC dedifferentiation in human aorta, making our findings a significant contribution to understanding its molecular limitations in MFS TAA. Concerning the proteolytic profile, carvedilol induced a significant upregulation of both MMP-9 and TIMP-1 protein expression, suggesting that it could trigger a broad activation of the ECM regulatory system, which nevertheless maintained a stable MMP-9/TIMP-1 ratio. This finding aligns with conflicting evidence in the literature already reported above. Given its non-selective nature, further studies are required to evaluate whether more selective β-blockers might exert different effects on those molecular pathways.

To further explore the potential of pharmacological synergy, we investigated in our model the effects of combined antihypertensive treatments. While ramipril and carvedilol monotherapies showed limited efficacy, their combination with losartan significantly improved the outcome by effectively reducing ED-A FN levels. Those results strongly support that the addition of losartan is essential to achieve a modulation of this marker. In relation to the proteolytic profile, our results demonstrate that losartan modulates remodeling markers differently depending on the specific drug combination. Specifically, when losartan was combined with carvedilol, we observed a notable reduction in both MMP-9 and TIMP-1 levels compared to carvedilol monotreatment. This stabilization likely results from the complementary action of these drugs: while carvedilol reduces hemodynamic wall stress, losartan antagonizes the AT1R/TGFβ signaling axis, which is the primary driver of protease overexpression in MFS aortopathy [32]. This synergy suggests that losartan can ‘correct’ the broad matrix activation previously induced by beta-blockers alone, restoring a more favorable proteolytic environment.

In contrast, the combination of losartan and ramipril paradoxically led to a significant upregulation of MMP-9, increasing the MMP-9/TIMP-1 ratio. This pro-proteolytic shift following a total blockade of the Renin-Angiotensin System (RAS) has been documented in other vascular models and may be attributed to the ‘Angiotensin II escape’ or the redirection of signaling toward the AT2 receptor. Indeed, studies have shown that dual RAS inhibition can increase local bradykinin levels and AT2R activation, which in certain pathological contexts can stimulate, rather than inhibit, MMP activity [33]. Furthermore, clinical evidence from the ONTARGET trial has highlighted that combining ACE inhibitors and ARBs does not necessarily provide superior tissue protection and may even lead to adverse remodeling responses compared to monotreatment [34]. In our experimental model, the specific inhibition of miR-632 emerged as a new potent therapeutic strategy, consistently outperforming conventional pharmacological drugs. While losartan acts upstream by antagonizing the AT1 receptor and indirectly modulating TGFβ signaling, miR-632 inhibition directly targets downstream molecular effectors directly involved in VSMC phenotypic switching and ECM remodeling.

Notably, miR-632 inhibition demonstrated a greater efficacy compared to losartan, as it not only suppressed its own expression and ED-A FN levels more effectively but also significantly reduced MMP-9 and TIMP-1 protein expression, thereby restoring a favorable proteolytic balance. Those results suggest that miR-632 plays a central regulatory role in the MFS aortopathy, acting as a master downstream effector of the TGFβ pathway. Consequently, targeting miR-632 may represent a more specific and possible therapeutic approach than standard pharmacological therapies.

## 5. Limitations

Finally, several limitations of this study must be acknowledged. First, all drugs were tested at a single concentration (10 µM) without dose-dependent kinetics. While this micromolar range is a standard screening approach to establish a sufficient diffusion gradient through the dense extracellular matrix of three-dimensional ex vivo aortic fragments, it does not reflect the complex pharmacokinetics used in clinical practice. Second, our model lacks the systemic physiology and hemodynamics of an in vivo organism. Additionally, the irregular geometry of the surgically excised tissue fragments mechanically precluded the execution of direct organ-level or biomechanical functional assays (such as wire myography or tensile testing), limiting our functional readouts to downstream molecular and biochemical signaling pathways. Third, the sample size (n = 6) is relatively small; however, this is justified by the preclinical nature of this mechanistic ex vivo study, focused on molecular and biochemical endpoints (RT-qPCR and Western blot), and the inherent rarity of fresh human aortic samples available from a single surgical center. To mitigate this constraint, a robust paired experimental design allowed each donor tissue to serve as its own internal control. Furthermore, our acute ex vivo TGFβ1-induction approach utilizes non-MFS tissues and cannot fully replicate the lifelong genetic and structural matrix alterations caused by intrinsic FBN1 mutations. Finally, because our experiments were conducted on advanced, surgically excised aneurysmal tissues, they do not preclude the possibility that losartan might exert more significant protective effects if administered during the earlier phases of MFS aortopathy. Therefore, future preclinical validation in chronic, patient-derived MFS tissue or in vivo genetic models is strictly necessary to confirm these therapeutic trends before clinical translation. Consequently, our conclusions regarding combined therapies and miR-632 inhibition must be interpreted cautiously within the boundaries of this ex vivo screening platform, serving as a promising mechanistic proof-of-concept rather than a definitive clinical outlook.

## 6. Conclusions

In conclusion, our study suggests losartan as the most effective conventional drug for reducing fibrotic markers in this ex vivo MFS model. Notably, our data also suggest that caution is required regarding combined pharmacological treatments, since certain drug combinations may paradoxically attenuate losartan efficacy or negatively affect the aortic proteolytic balance. Furthermore, miR-632 inhibition showed a promising capacity to counteract aortic remodeling in this ex vivo model of MFS TAA, although extensive future in vivo validation remains strictly necessary to evaluate its safety and actual therapeutic potential. Overall, these preclinical findings emphasize the importance of choosing a meticulously targeted therapeutic approach to optimize the management of aortic wall degeneration and, prospectively, delay the progression of thoracic aortic aneurysms in MFS patients.

## Figures and Tables

**Table 1:** Experimental setup and sample allocation for molecular analysis.

Experimental Parameter	Gene Expression Analysis (RNA)	Protein Expression Analysis
Enrolled Patients	(n = 6 non-MFS TAA cohort)	(n = 6 non-MFS TAA same cohort)
Culture System	24-well multi-well plates	6-well multi-well plates
Fragments per Patient	3 uniform aortic fragments	5 uniform aortic fragments
Total Tissue Fragments	18 fragments per treatment group	30 fragments per treatment group
Experimental Arms	TGF-β, losartan, ramipril, carvedilol, 2 combinations, scramble, scramble + Losartan and miR-632 Inhibitor	TGF-β, losartan, ramipril, carvedilol, 2 combinations, scramble, scramble + Losartan and miR-632 Inhibitor
Incubation Time	24 h	72 h
Post-Treatment Pooling	2 Pools of 3 patients each (Pool A & Pool B)	2 Pools of 3 patients each (Pool A & Pool B)
Analytical Technique	Real-Time Quantitative PCR (RT-qPCR)	Western Blotting

## Data Availability

The data are contained within the article or in Supplementary Table S1.

## Supplementary Materials

The following supporting information can be downloaded at https://www.mdpi.com/article/10.3390/biom16060863/s1. Table S1: Primer sequences used for real-time PCR. File S1: Original western blots.

## References

[1] Salmasi M.Y., Alwis S., Cyclewala S., Jarral O.A., Mohamed H., Mozalbat D., Nienaber C.A., Athanasiou T., Morris-Rosendahl D. (2023). Members of the London Aortic Mechanobiology Working Group. The genetic basis of thoracic aortic disease: The future of aneurysm classification?. Hell. J. Cardiol..

[2] Dawson A., Li Y., Li Y., Ren P., Vasquez H.G., Zhang C., Rebello K.R., Ageedi W., Azares A.R., Mattar A.B. (2021). Single-Cell Analysis of Aneurysmal Aortic Tissue in Patients with Marfan Syndrome Reveals Dysfunctional TGF-β Signaling. Genes.

[3] Zeigler S.M., Sloan B., Jones J.A. (2021). Pathophysiology and Pathogenesis of Marfan Syndrome. Adv. Exp. Med. Biol..

[4] Arif R., Zaradzki M., Remes A., Seppelt P., Kunze R., Schröder H., Schwill S., Ensminger S.M., Robinson P.N., Karck M. (2017). AP-1 Oligodeoxynucleotides Reduce Aortic Elastolysis in a Murine Model of Marfan Syndrome. Mol. Ther. Nucleic Acids.

[5] Romaniello F., Mazzaglia D., Pellegrino A., Grego S., Fiorito R., Ferlosio A., Chiariello L., Orlandi A. (2014). Aortopathy in Marfan syndrome: An update. Cardiovasc. Pathol..

[6] Wolosowicz M., Prokopiuk S., Kaminski T.W. (2025). Matrix Metalloproteinase-9 (MMP-9) as a Therapeutic Target: Insights into Molecular Pathways and Clinical Applications. Pharmaceutics.

[7] Chiu H.H. (2021). An update of medical care in Marfan syndrome. Tzu Chi Med. J..

[8] Bin Mahmood S.U., Velasquez C.A., Zafar M.A., Saeyeldin A.A., Brownstein A.J., Ziganshin B.A., Elefteriades J.A., Mukherjee S.K. (2017). Medical management of aortic disease in Marfan syndrome. Ann. Cardiothorac. Surg..

[9] Li-Wan-Po A., Loeys B., Farndon P., Latham D., Bradley C. (2011). Preventing the aortic complications of Marfan syndrome: A case-example of translational genomic medicine. Br. J. Clin. Pharmacol..

[10] Elbadawi A., Omer M.A., Elgendy I.Y., Abuzaid A., Mohamed A.H., Rai D., Saad M., Mentias A., Rezq A., Kamal D. (2019). Losartan for Preventing Aortic Root Dilatation in Patients with Marfan Syndrome: A Meta-Analysis of Randomized Trials. Cardiol. Ther..

[11] Sica D.A., Halstenson C.E., Gehr T.W., Keane W.F. (2000). Pharmacokinetics and blood pressure response of losartan in end-stage renal disease. Clin. Pharmacokinet..

[12] D’Amico F., Doldo E., Pisano C., Scioli M.G., Centofanti F., Proietti G., Falconi M., Sangiuolo F., Ferlosio A., Ruvolo G. (2020). Specific miRNA and Gene Deregulation Characterize the Increased Angiogenic Remodeling of Thoracic Aneurysmatic Aortopathy in Marfan Syndrome. Int. J. Mol. Sci..

[13] Terriaca S., Scioli M.G., Pisano C., Ruvolo G., Ferlosio A., Orlandi A. (2023). miR-632 Induces DNAJB6 Inhibition Stimulating Endothelial-to-Mesenchymal Transition and Fibrosis in Marfan Syndrome Aortopathy. Int. J. Mol. Sci..

[14] Evangelista A., Flachskampf F.A., Erbel R., Antonini-Canterin F., Vlachopoulos C., Rocchi G., Sicari R., Nihoyannopoulos P., Zamorano J., European Association of Echocardiography (2010). Echocardiography in aortic diseases: EAE recommendations for clinical practice. Eur. J. Echocardiogr..

[15] Fitzpatrick E., Han X., Liu W., Corcoran E., Burtenshaw D., Morrow D., Helt J.C., Cahill P.A., Redmond E.M. (2017). Alcohol Reduces Arterial Remodeling by Inhibiting Sonic Hedgehog-Stimulated Stem Cell Antigen-1 Positive Progenitor Stem Cell Expansion. Alcohol Clin. Exp. Res..

[16] Wilson D.P., Saward L., Zahradka P., Cheung P.K. (1999). Angiotensin II receptor antagonists prevent neointimal proliferation in a porcine coronary artery organ culture model. Cardiovasc. Res..

[17] Lunder M., Janić M., Žiberna L., Drevenšek G., Šabovič M. (2012). A low-dose atorvastatin and losartan combination directly improves aortic ring relaxation and diminishes ischaemic-reperfusion injury in isolated rat hearts. Med. Sci. Monit..

[18] Yang G., Zhang Z., Ma X., Chen J., Shi H., Yang J., Han Q. (2025). Role of Carvedilol in Inhibiting the Proliferation and Migration of Vascular Smooth Muscle Cells by Upregulating microRNA-145 Expression. Physiol. Res..

[19] Macabrey D., Deslarzes-Dubuis C., Longchamp A., Lambelet M., Ozaki C.K., Corpataux J.M., Allagnat F., Déglise S. (2022). Hydrogen Sulphide Release via the Angiotensin Converting Enzyme Inhibitor Zofenopril Prevents Intimal Hyperplasia in Human Vein Segments and in a Mouse Model of Carotid Artery Stenosis. Eur. J. Vasc. Endovasc. Surg..

[20] Terriaca S., Scioli M.G., Bertoldo F., Pisano C., Nardi P., Balistreri C.R., Magro D., Belmonte B., Savino L., Ferlosio A. (2024). miRNA-Driven Regulation of Endothelial-to-Mesenchymal Transition Differs among Thoracic Aortic Aneurysms. Cells.

[21] Livak K.J., Schmittgen T.D. (2001). Analysis of relative gene expression data using real-time quantitative PCR and the 2(-Delta Delta C(T)) Method. Methods.

[22] Sellers S.L., Milad N., Chan R., Mielnik M., Jermilova U., Huang P.L., de Crom R., Hirota J.A., Hogg J.C., Sandor G.G. (2018). Inhibition of Marfan Syndrome Aortic Root Dilation by Losartan: Role of Angiotensin II Receptor Type 1-Independent Activation of Endothelial Function. Am. J. Pathol..

[23] Miguel-Carrasco J.L., Beaumont J., San José G., Moreno M.U., López B., González A., Zalba G., Díez J., Fortuño A., Ravassa S. (2017). Mechanisms underlying the cardiac antifibrotic effects of losartan metabolites. Sci. Rep..

[24] Patil N., Patil V.S., Punase N., Mapare G., Bhatt S., Patil C.R. (2025). Comparative Efficacy of β-Carotene and Losartan Against Isoproterenol-Induced Cardiac Fibrosis: An Experimental and Computational Studies. J. Am. Nutr. Assoc..

[25] Fang Q.Q., Wang X.F., Zhao W.Y., Ding S.L., Shi B.H., Xia Y., Yang H., Wu L.H., Li C.Y., Tan W.Q. (2018). Angiotensin-converting enzyme inhibitor reduces scar formation by inhibiting both canonical and noncanonical TGF-β1 pathways. Sci. Rep..

[26] Wylie-Sears J., Levine R.A., Bischoff J. (2014). Losartan inhibits endothelial-to-mesenchymal transformation in mitral valve endothelial cells by blocking transforming growth factor-β-induced phosphorylation of ERK. Biochem. Biophys. Res. Commun..

[27] Dell’Italia L.J., Collawn J.F., Ferrario C.M. (2018). Multifunctional Role of Chymase in Acute and Chronic Tissue Injury and Remodeling. Circ. Res..

[28] Nakaya M., Chikura S., Watari K., Mizuno N., Mochinaga K., Mangmool S., Koyanagi S., Ohdo S., Sato Y., Ide T. (2012). Induction of cardiac fibrosis by β-blocker in G protein-independent and G protein-coupled receptor kinase 5/β-arrestin2-dependent Signaling pathways. J. Biol. Chem..

[29] Okumura K., Kato H., Honjo O., Breitling S., Kuebler W.M., Sun M., Friedberg M.K. (2015). Carvedilol improves biventricular fibrosis and function in experimental pulmonary hypertension. J. Mol. Med..

[30] Jordan M., Schmidt K., Fuchs M., Just A., Pfanne A., Willmer L., Neubert L., Werlein C., Zardo P., Pich A. (2025). Repurposing of the small-molecule adrenoreceptor-inhibitor carvedilol for treatment of the fibrotic lung. Front. Pharmacol..

[31] El-Wakeel S.A., Rahmo R.M., El-Abhar H.S. (2018). Anti-fibrotic impact of Carvedilol in a CCl-4 model of liver fibrosis via serum microRNA-200a/SMAD7 enhancement to bridle TGF-β1/EMT track. Sci. Rep..

[32] Habashi J.P., Judge D.P., Holm T.M., Cohn R.D., Loeys B.L., Cooper T.K., Myers L., Klein E.C., Liu G., Calvi C. (2006). Losartan, an AT1 antagonist, prevents aortic aneurysm in a mouse model of Marfan syndrome. Science.

[33] Romero C.A., Mathew S., Wasinski B., Reed B., Brody A., Dawood R., Twiner M.J., McNaughton C.D., Fridman R., Flack J.M. (2021). Angiotensin-converting enzyme inhibitors increase anti-fibrotic biomarkers in African Americans with left ventricular hypertrophy. J. Clin. Hypertens..

[34] Unger T. (2003). The ongoing telmisartan alone and in combination with ramipril global endpoint trial program. Am. J. Cardiol..

