# Supplementary material for: The Efficacy of Antihypertensive Drugs and miR-632 Inhibition on Parietal Remodeling in a Model of Marfan Thoracic Aortic Aneurysm
Source: Biomolecules. 2026 Jun 12;16(6):863. doi: 10.3390/biom16060863 (PMC13296651; doi:10.3390/biom16060863)
Supplement: Supplementary file 1 [file biomolecules-16-00863-s001.zip › biomolecules-4341464 original WB image .pdf]

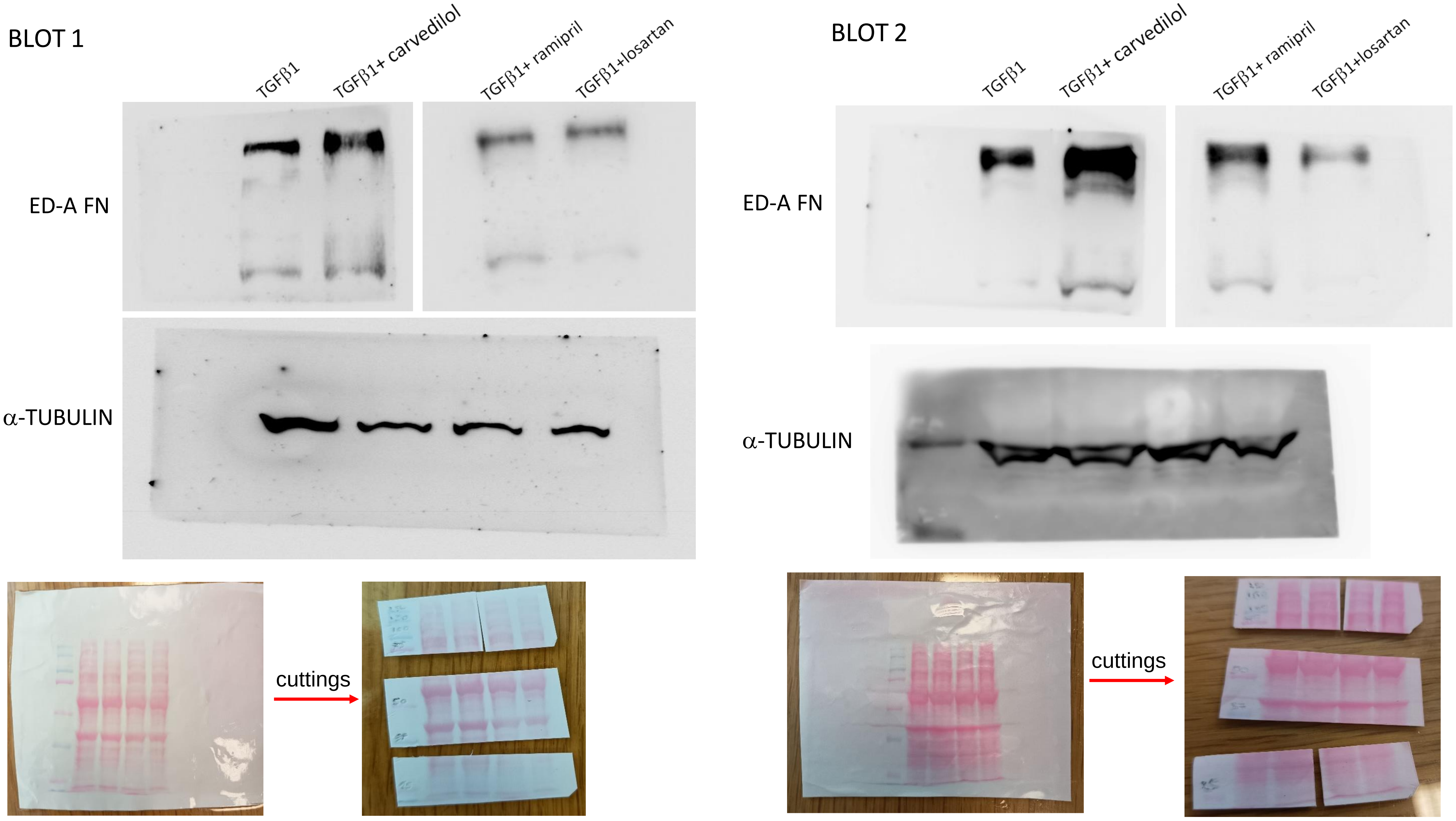
BLOT 1
TGFβ1
TGFβ1+ carvedilol
TGFβ1+ ramipril
TGFβ1+losartan
ED-A FN
α-TUBULIN
cuttings
BLOT 2
TGFβ1
TGFβ1+ carvedilol
TGFβ1+ ramipril
TGFβ1+losartan
ED-A FN
α-TUBULIN
cuttings

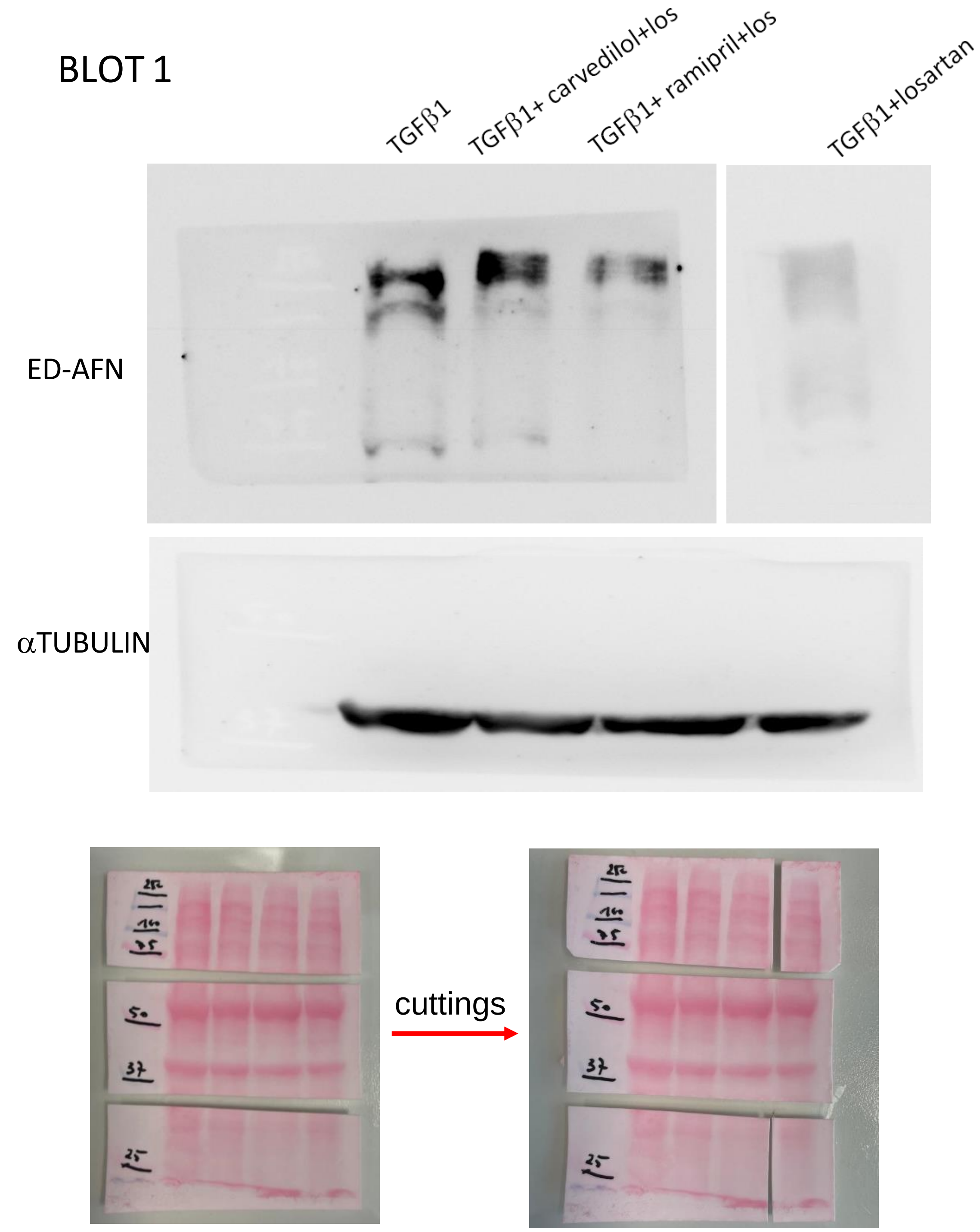
BLOT 1
TGFβ1
TGFβ1+ carvedilol+los
TGFβ1+ ramipril+los
TGFβ1+losartan
ED-AFN
αTUBULIN
cuttings

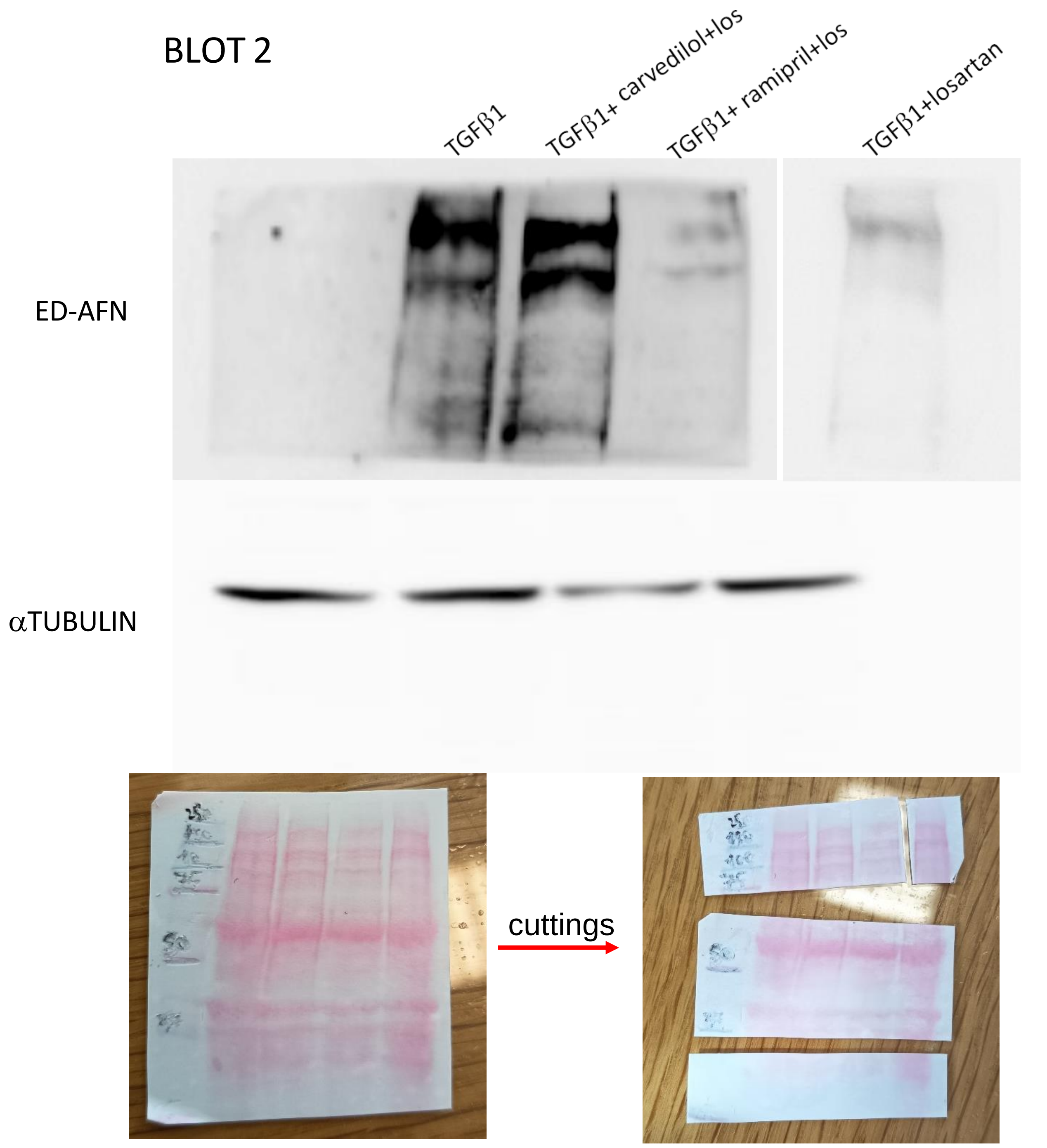
BLOT 2
TGFβ1
TGFβ1+ carvedilol+los
TGFβ1+ ramipril+los
TGFβ1+losartan
ED-AFN
αTUBULIN
cuttings

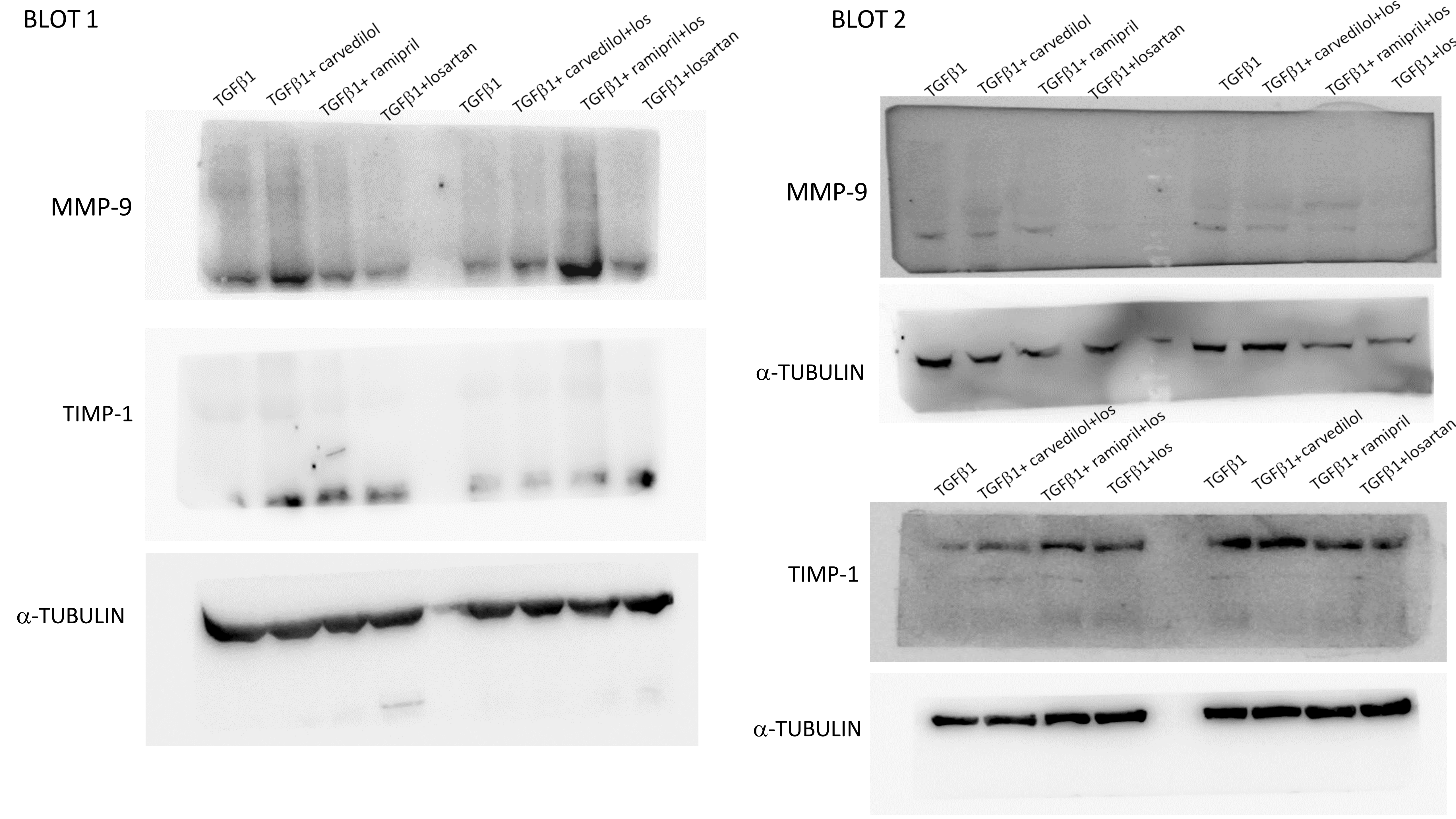
BLOT 1
TGFβ1
TGFβ1+ carvedilol
TGFβ1+ ramipril
TGFβ1+losartan
TGFβ1
TGFβ1+ carvedilol+los
TGFβ1+ ramipril+los
TGFβ1+losartan
MMP-9
TIMP-1
α-TUBULIN
BLOT 2
TGFβ1
TGFβ1+ carvedilol
TGFβ1+ ramipril
TGFβ1+losartan
TGFβ1
TGFβ1+ carvedilol+los
TGFβ1+ ramipril+los
TGFβ1+los
MMP-9
α-TUBULIN
TGFβ1
TGFβ1+ carvedilol+los
TGFβ1+ ramipril+los
TGFβ1+los
TGFβ1
TGFβ1+carvedilol
TGFβ1+ ramipril
TGFβ1+losartan
TIMP-1
α-TUBULIN

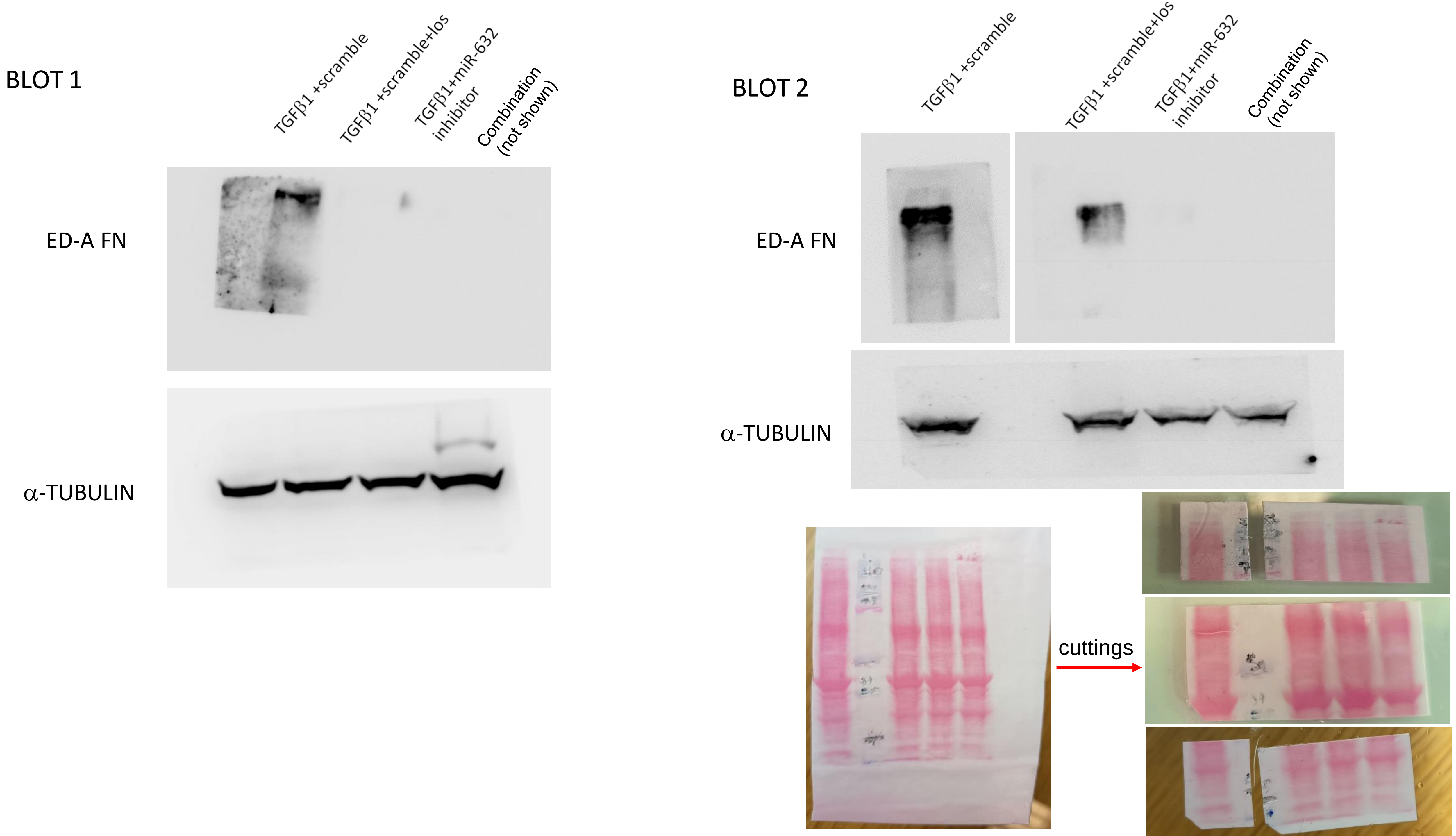
BLOT 1
TGFβ1 +scramble
TGFβ1 +scramble+los
TGFβ1+miR-632 inhibitor
Combination (not shown)
ED-A FN
α-TUBULIN
BLOT 2
TGFβ1 +scramble
TGFβ1 +scramble+los
TGFβ1+miR-632 inhibitor
Combination (not shown)
ED-A FN
α-TUBULIN
cuttings

BLOT 1

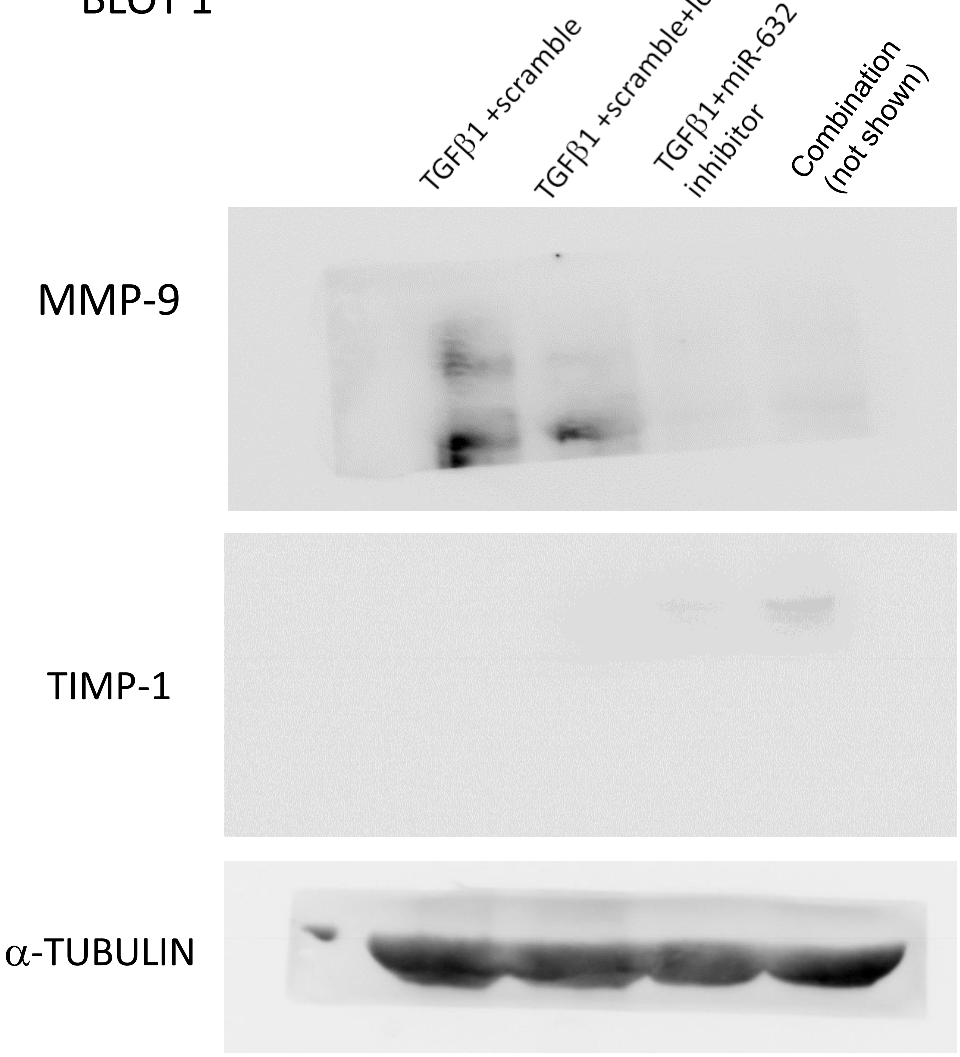
TGFβ1 +scramble
TGFβ1 +scramble+los
TGFβ1+miR-632 inhibitor
Combination (not shown)
MMP-9
TIMP-1
α-TUBULIN

BLOT 2

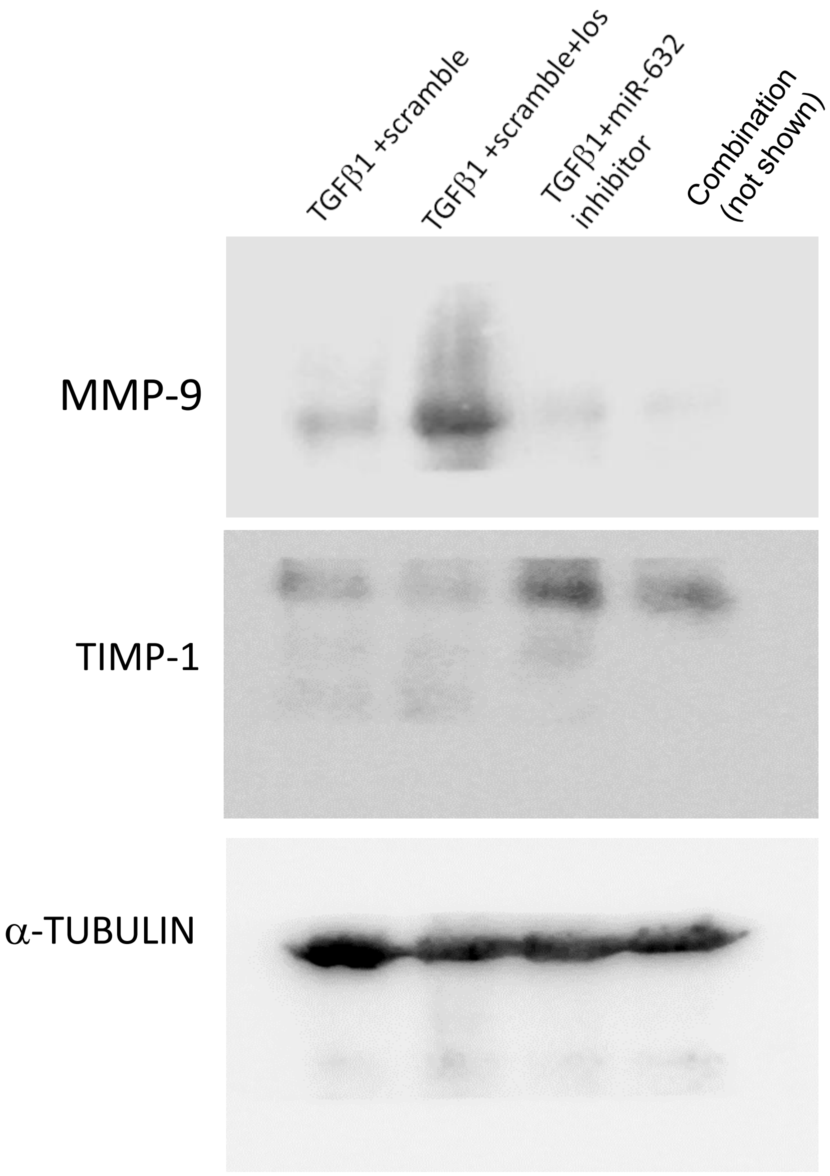
TGFβ1 +scramble
TGFβ1 +scramble+los
TGFβ1+miR-632 inhibitor
Combination (not shown)
MMP-9
TIMP-1
α-TUBULIN
