# Supplementary material for: The Efficacy of Antihypertensive Drugs and miR-632 Inhibition on Parietal Remodeling in a Model of Marfan Thoracic Aortic Aneurysm
Source: Biomolecules. 2026 Jun 12;16(6):863. doi: 10.3390/biom16060863 (PMC13296651; doi:10.3390/biom16060863)
Supplement: Supplementary file 1 [file biomolecules-16-00863-s001.zip › biomolecules-4341464-supplementary.pdf]

Supplemental Table S1. Primer sequences used for Real-time PCR

GENE/miRNA	PRIMER SEQUENCES/Cat. Number
MystiCq microRNA hsa-miR-632	MIRAP00623 (Merk KGaA, Darmstadt, Germany)
h-RNU6-1	Forward: 5’-CTCGCTTCGGCAGCACA-3’
Universal PCR Reverse Primer	Cat. N° 54410 (Norgen Biotek, Thorold, Canada)
h- ED-A FN	Forward: 5’-CCAGTCCACAGCTATTCCTG-3’<br>Reverse: 5’-ACAACCACGGATGAGCTG-3’
h-GAPDH	Forward: 5’-ACGGATTTGGTCGTATTGG-3’<br>Reverse: 5’-GATTTTGGAGGGATCTCGC-3’
